# Improving high-resolution copy number variation analysis from next generation sequencing using unique molecular identifiers

**DOI:** 10.1186/s12859-021-04060-4

**Published:** 2021-03-12

**Authors:** Pierre-Julien Viailly, Vincent Sater, Mathieu Viennot, Elodie Bohers, Nicolas Vergne, Caroline Berard, Hélène Dauchel, Thierry Lecroq, Alison Celebi, Philippe Ruminy, Vinciane Marchand, Marie-Delphine Lanic, Sydney Dubois, Dominique Penther, Hervé Tilly, Sylvain Mareschal, Fabrice Jardin

**Affiliations:** 1 INSERM U1245, Team Genomics and Biomarkers of Lymphoma and Solid Tumors, Normandie Univ, UNIROUEN, Rouen, France; 2 Centre Henri Becquerel, Rouen, France; 3 Master Bioinformatique BIM, Normandie Univ, UNIROUEN, Rouen, France; 4 LITIS EA 4108, Normandie Univ, UNIROUEN, Rouen, France; 5 LMRS UMRS 6085, Normandie Univ, UNIROUEN, Rouen, France; 6 INSERM U1052 UMR CNRS 5286, Cancer Research Center of Lyon, Lyon, France

**Keywords:** UMI, CNV calling, Next generation sequencing

## Abstract

**Background:**

Recently, copy number variations (CNV) impacting genes involved in oncogenic pathways have attracted an increasing attention to manage disease susceptibility. CNV is one of the most important somatic aberrations in the genome of tumor cells. Oncogene activation and tumor suppressor gene inactivation are often attributed to copy number gain/amplification or deletion, respectively, in many cancer types and stages. Recent advances in next generation sequencing protocols allow for the addition of unique molecular identifiers (UMI) to each read. Each targeted DNA fragment is labeled with a unique random nucleotide sequence added to sequencing primers. UMI are especially useful for CNV detection by making each DNA molecule in a population of reads distinct.

**Results:**

Here, we present molecular Copy Number Alteration (mCNA), a new methodology allowing the detection of copy number changes using UMI. The algorithm is composed of four main steps: the construction of UMI count matrices, the use of control samples to construct a pseudo-reference, the computation of log-ratios, the segmentation and finally the statistical inference of abnormal segmented breaks. We demonstrate the success of mCNA on a dataset of patients suffering from Diffuse Large B-cell Lymphoma and we highlight that mCNA results have a strong correlation with comparative genomic hybridization.

**Conclusion:**

We provide mCNA, a new approach for CNV detection, freely available at https://gitlab.com/pierrejulien.viailly/mcna/ under MIT license. mCNA can significantly improve detection accuracy of CNV changes by using UMI.

**Supplementary Information:**

The online version contains supplementary material available at 10.1186/s12859-021-04060-4.

## Background

Recently, copy number variations (CNV) impacting genes involved in oncogenic pathways have attracted an increasing attention to manage disease susceptibility [1, 2]. CNV is one of the most important somatic aberrations in the genome of tumor cells. Oncogene activation and tumor suppressor gene inactivation are often attributed to copy number gain/amplification or deletion, respectively, in many cancer types and stages.

CNV analysis refers to the detection of a difference in the dosage of a genomic locus containing one or more dosage-sensitive genes (zygosity). The resolution limit of conventional cytogenetics (approximately 5 Mb) has been improved by molecular cytogenetics using comparative genomic hybridization (CGH) and more recently array comparative genomic hybridization (aCGH). These technologies make it possible to detect genomic imbalances of ${<100}$ kb, whereas more specialized array designs increase the resolution to $\le 200$ bp for specific targeted regions. Despite these performances, aCGH requires the purchase of a specific platform for data acquisition and its resolution is limited to the detection of tumoral clones that differ substantially in DNA content from a reference.

Next Generation Sequencing technologies (NGS) have rapidly supplanted traditional Sanger sequencing as the preferred methodology for the detection of actionable single nucleotide variations (SNV) in oncology. Diagnostic laboratories are now massively equipped with Illumina/Thermofisher sequencers. Massively parallel sequencing offers many advantages including high sensitivity and specificity for SNV and CNV detection within a single platform. Nevertheless, libraries must be amplified by PCR to produce a sufficient amount of signal. This amplification step introduces many biases for counting reads because the number of produced reads is no longer directly proportional to the number of initial unique targeted DNA fragments. The amplification factor of each region is unknown and depends on many parameters such as library size, GC content, region length or competition between primers overlapping the same locus while using amplicon-based libraries.

There are three main approaches to identify CNV from NGS data: read-pair (RP), split-read (SR), and read-depth (RD).

RP methods (BreakDancer [3], PEMer [4], Ulysses [5]) consist in comparing the average insert size between the sequenced read-pairs with an expected size based on a reference genome. The discordance between mapped paired-reads and the predetermined average insert size is then used to identify gain and loss of materials. Shorter/longer insert size than expected will correlate to the loss/gain of material, respectively.

SR methods evaluate CNV using paired reads where only one read of the pair has a reliable mapping quality whereas the other one partially fails to map to the reference sequence. These discrepancies within a read pair can potentially provide the precise position of insertion/deletion events. Several tools implementing SR strategies enable the detection of these breakpoints (SVseq2 [6], Gustaf [7], PRISM [8]) but they are limited to short insertions or deletions.

The RD approach consists in counting aligned reads overlapping a genomic region in a sliding window. These read counts (RC) are then compared between the sample of interest and a reference to compute CNV segmentation. A local decrease in sequencing depth will be associated with a loss of genomic material whereas its increase will be correlated to locus gain/amplification. Several tools were developed using RD-based approaches (CNVnator [9], CNV-seq [10]). This strategy seems particularly promising for the analysis of targeted sequencing experiments (TSE). TSE enables the sequencing of key genes or regions of interest to high depth (500–1000X or higher) and provides a cost-effective strategy to identify variants at low allele frequencies. Some tools, such as ONCOCNV [11], were specially developed for the analysis of targeted amplicon-based libraries. Many biases due to the amplification step while preparing this type of library prevent the direct quantification of loci copy-number (size of the library, GC percentage, amplicon length, primer melting temperature, competition between primers...). It implies the use of normalization strategies to allow the comparison of read counts between samples.

Recent advances in NGS protocols allow for the addition of unique molecular identifiers (UMI) to each read. Each targeted DNA fragment is labeled by a unique random nucleotide sequence added to sequencing primers. UMI are especially useful for CNV detection by making each DNA molecule in a population of reads distinct. They allow the direct count of targeted DNA molecules before the library amplification by simply counting the number of unique UMI sequences per position of the alignment.

Here, we present mCNA (molecular Copy Number Alteration), a new methodology allowing the detection of copy number changes using UMI. We demonstrate the success of our algorithm on a dataset of patients diagnosed with Diffuse Large B-cell Lymphoma (DLBCL) and we highlight that mCNA results have a strong correlation with CGH. To assess the robustness and sensitivity limit of our approach, we used in silico simulation of copy number aberrations in a control sample and also sequential dilutions of REC-1 cell line.

## Methods

### Library construction

A Pan-lymphoma panel was designed using the QIAseq Targeted DNA Custom Panel Builder (QIAGEN) to identify alterations within important genes for lymphomagenesis. This panel targets 69 genes (hotspots, regions or whole gene) using 1493 gene specific primers. List of genes and number of GSP per gene are provided in Additional file 1: Table S1.

**Fig. 1:**
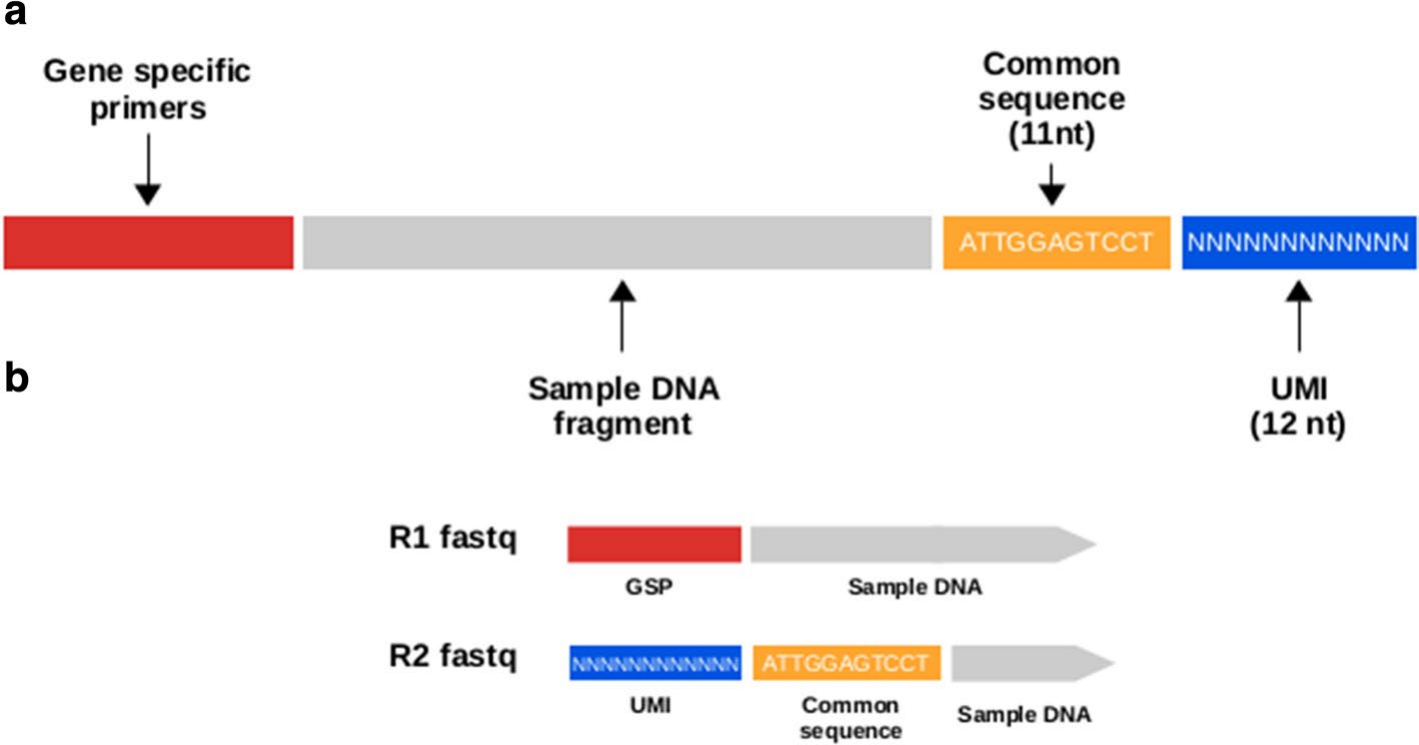
Qiaseq library construction scheme. **a** Libraries were composed of a pool of gene specific primers (GSP) designed to target genomic regions of interest, a sample DNA fragment, a common sequence of 11 nucleotides used during the library construction as an universal primer and finally a random sequence of 12 nucleotides (UMI). **b** Structure of reads in the Fastq files obtained after paired-end sequencing

The QIAseq Targeted DNA chemistry introduces molecular barcodes (UMI) to enable digital sequencing and to identify PCR duplicates (Fig. 1). The molecular barcodes are short aleatory nucleotide sequences of 12 bp length added to each read before the library amplification. Statistically, this process provides $4^{12}$ possible indices per adapter; hence, each DNA molecule in the sample receives a unique UMI sequence.

### Subjects and methods

#### Study design and patients

22 adult patients with de novo CD20+ Diffuse Large B-cell Lymphoma (DLBCL) or primary mediastinal B-cell lymphoma (PMBL) were selected from the prospective, multicenter, and randomized LNH-03B LYSA trials with available frozen tumor samples and adequate DNA quality. CGH was previously performed for these samples after whole-genome amplification against a Promega normal DNA pool using Agilent SurePrint G3 $4\times 180$ K microarrays. Briefly, arrays were scanned with Agilent Feature Extraction and processed with cghRA pipeline as previously described [12].

DNA from REC-1 cell line, established from the lymph node of a 61-year-old man with refractory B-cell lymphoma, was extracted. Dilutions at 50%, 30%, 20%, 10% and 5% of this DNA were performed using Human Mixed Genomic DNA Promega. Human Genomic DNA comes from multiple anonymous donors.

Five blood samples of healthy individuals were collected and used as a control to construct the pseudo-reference profile.

#### Sample collection and sequencing

Tumor genomic DNA (gDNA) was isolated from fresh diagnostic tissue biopsies or blood. Samples were quantified using QuBit High Sensitivity dsDNA (Thermo Fisher Scientific).

gDNA samples were sequenced with the entire Pan-lymphoma panel. 30 ng of gDNA were enzymatically fragmented and end repaired, followed by ligation of the molecular barcoded adaptators (UMI). After purification, target enrichment was carried out using the set of 1493 gene specific primers. Then, enriched DNA was submitted to universal PCR with a number of cycle adapted to this number of primers. Purified libraries were quantified using QuBit High Sensitivity dsDNA.

Finally, libraries were sequenced on Illumina MiSeq (paired-end, $2\times 150$ bp) following manufacturer’s user manual (Illumina, CA).

#### Library sequencing and bioinformatics pre-processing

Briefly, gene-specific primers and common regions were trimmed from R1 and R2 fastq using an in-house program. UMI sequences were extracted from read construction using UMI-tools [13].

Reads were aligned against hg19 reference genome using BWA-mem [14] and standardized according to the GATK3 Best Practices recommendations. A detailed bioinformatics pipeline is provided in Additional file 1: Fig. S1.

## mCNA algorithm

**Fig. 2:**
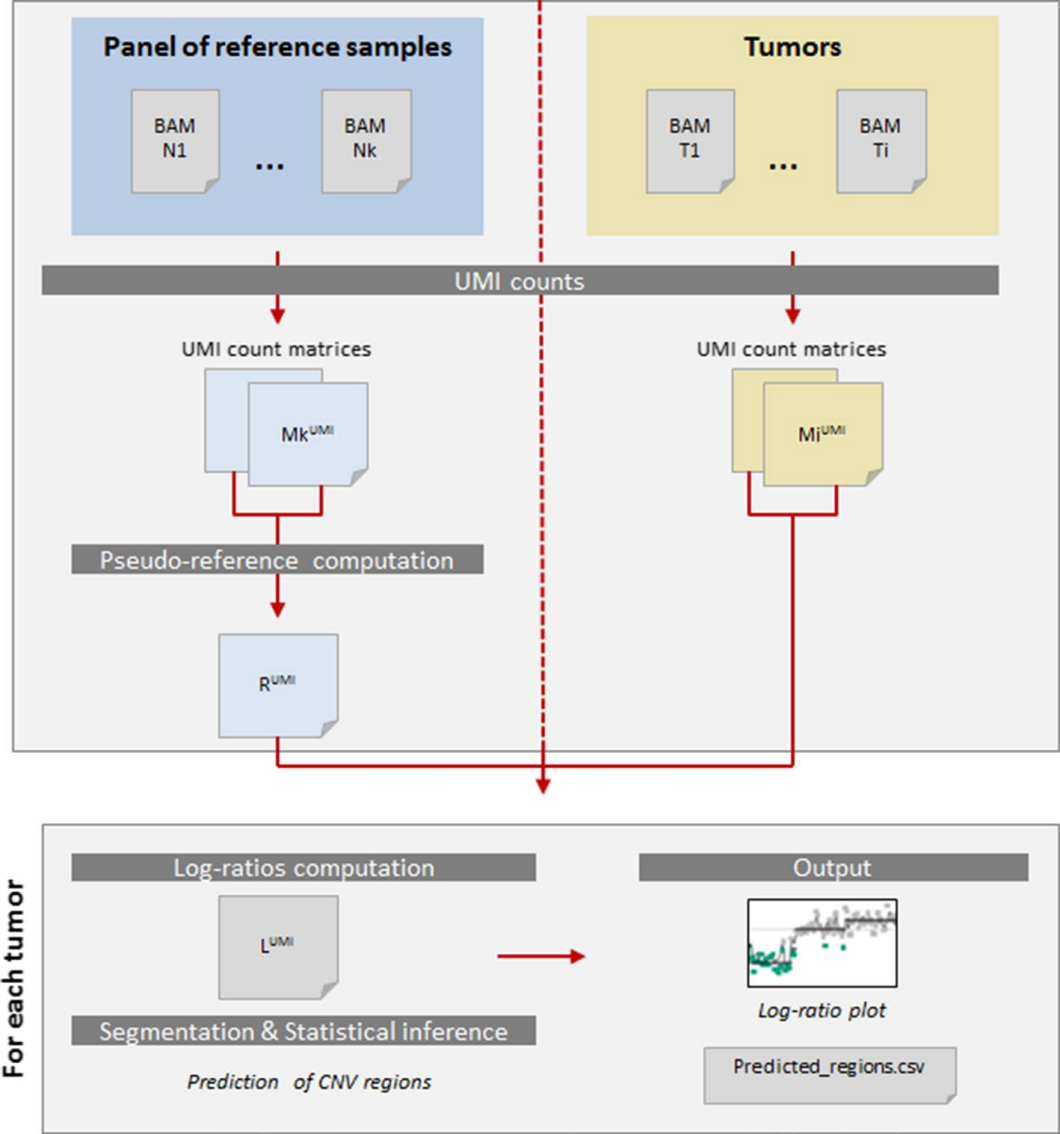
Workflow of mCNA data processing. Processing of control samples: Matrices of UMI counts are constructed by counting number of UMI overlapping each targeted region from the BED file. Then, each value of both matrices are divided respectively by the mean UMI-depth to standardize sample counts. A pseudo-reference is constructed giving for each region the geometric mean of observed UMI-depth. Processing of tumoral samples: Using the same processes than for reference samples, UMI-depth matrices are computed and normalized. Log-ratios computing and segmentation: log-ratios are computed and segmentation is then performed for each gene by CBS. Statistical tests are performed to determine whether observed signals within segments are significantly distinct from 0

In this article, we present a new strategy to detect copy number changes for targeted panels of genes using UMI. The algorithm is composed of four main steps: the construction of UMI count matrices, the use of control samples to construct a pseudo-reference, the computation of log-ratios (LR), the segmentation and finally the statistical inference of abnormal segmented breaks (Fig. 2).

### Prerequisites

mCNA algorithm requires sequencing libraries introducing one or more short aleatory sequences (Unique Molecular Identifiers, UMI) in reads construction. UMI sequences must be extracted from raw FASTQ files before alignment and appended to read identifiers using UMI-tools [13]. Processed reads must be aligned against a reference genome to produce BAM file. A BED file is also required, giving for each targeted region the chromosome name, the start/end positions of the locus and the gene name.

Details for complete bioinformatics processing of QIAseq Targeted DNA Panel are provided in Additional file 1: Fig. S1.

### Construction of UMI-depth matrices

We define $M^{\text {UMI}}$ as the UMI-depth matrix of one BAM file. $P$ is the total number of targeted regions. $C_{\text {p}}^{\text {UMI}}$ reflects the number of unique UMI overlapping $p$ region and $U$ the total number of unique UMI of one sample.

Each region supplied in the BED file is scanned using *scanBam* function of Rsamtools package [15]. $C_{\text {p}}^{\text {UMI}}$ is computed from unique UMI sequences extracted from read names overlapping $p$.

Each matrix $M^{\text {UMI}}$ is finally normalized by $U$ to allow the comparison between samples, as shown in the Additional file 1: Fig S2.

### Pseudo-reference construction

From $M^{\text {UMI}}$ matrices of normal samples, a geometric mean of $C_{\text {p}}^{\text {UMI}}/U$ is computed line by line to create a vector $R^{\text {UMI}}$ of dimensions $(1,P)$.

To automatically detect outlier samples, Root-Mean-Square Deviations (RMSD) are computed between $C_{\text {p}}^{\text {UMI}}/U$ and $R_{\text {p}}^{\text {UMI}}$ for each region p of each control sample.

Samples with at least 20% of regions with $RMSD_{p} > T$ are excluded from baseline construction, with $T$ defined as:

$$\begin{aligned} T = Q3(RMSD_{p}) + 1.5 \times IQR(RMSD_{p}) \end{aligned}$$

If at least one sample is filtered, $R^{\text {UMI}}$ vector is updated with passing filter $M^{\text {UMI}}$ matrices only. The same process is applied to detect outlier noisy regions. These positions are defined as sequenced regions with at least $RMSD_{p} > T$ in 50% of control samples.

### Log-ratios and signal centering

We define the log-ratio $L_{\text {p}}^{\text {UMI}}$ as:

$$\begin{aligned} L_{\text {p}}^{\text {UMI}} = log2\left( \frac{M_{\text {p}}^{\text {UMI}}}{R_{\text {p}}^{\text {UMI}}} \right) \end{aligned}$$

where $M_{\text {p}}^{\text {UMI}}$ is the UMI count of a tumor sample for the region $p$ and $R_{\text {p}}^{\text {UMI}}$ is the UMI pseudo-reference vector of control samples for the region $p$.

A Gaussian mixture model with one to three mixture components is estimated from $L_{\text {p}}^{\text {UMI}}$ using *Mclust* function of R package mclust [16]. The estimated gaussian closest to $\widehat{L^{\text {UMI}}}=0$ is used to center the signal by subtracting its average from the $L_{\text {p}}^{\text {UMI}}$ values. Indeed, we assume that the Gaussian of our signal closest to 0 corresponds to a diploid state. This centering step could be disabled via the program’s arguments.

### Segmentation

Each gene is composed of *n* consecutive regions and we define a vector of log-ratios $V_{\text {n}}^{\text {UMI}}$ used for segmentation, as:

$$\begin{aligned} V_{\text {n}}^{\text {UMI}} = \left\{ L_{\text {p}}^{\text {UMI}} ; L_{\text {p+1}}^{\text {UMI}} ; \cdots {}\right\} (p \in n) \end{aligned}$$

mCNA uses the circular binary segmentation (CBS) method implemented in the R package PSCBS [17] to segment $V_{\text {n}}^{\text {UMI}}$.

To avoid breakpoints at outlier values, a vector of weights $W$ is given to CBS segmentation function. $W$ is inversely proportional to the variances of $C_{\text {p}}^{\text {UMI}}/U$ observed in the control samples and defined as:

$$\begin{aligned} W_{\text {p}}=\frac{1}{var(M_{\text {p}}^{\text {UMI}}/U)} ( \text {within controls}) \end{aligned}$$

${W_{\text {p}}}$ are then transformed to be limited to the interval [0,1] as follows:

$$\begin{aligned} W'_{\text {p}} = \frac{ W_{\text {p}} - min(W_{\text {p}}) }{ max(W_{\text {p}}) - min(W_{\text {p}}) } \end{aligned}$$

Finally, a Student’s t-test is performed on each segmented region to test whether or not the $V_{\text {n}}^{\text {UMI}}$ vector is significantly different from the reference value of 0. To avoid false positive segments, a FDR correction is applied.

### Estimation of tumoral content

We define $G_{\text {n}}^{\text {UMI}}$ and $D_{\text {n}}^{\text {UMI}}$ the vectors $V_{\text {n}}^{\text {UMI}}$ of significant amplified/deleted segments, respectively. We use $D_{\text {n}}^{\text {UMI}}$ and $G_{\text {n}}^{\text {UMI}}$ distributions to estimate tumor enrichment assuming that means of these distributions reflect a gain/loss of one segment copy and that log-ratios are a mixture of both tumoral and normal signals. We define as $c$ the percentage of tumor enrichment to estimate.

Two independent estimates of $c$ were produced: one from the significantly deleted segments and the other from those amplified. The estimation of $c$ cannot be done in one step because log-ratios involving one gain or one loss are not symmetrical. For example, the loss of one copy of a segment in a sample containing only tumor cells will lead to a log-ratio equal to $\log _2(\frac{1}{2})=-1$ while a gain of one copy will lead to $\log _2(\frac{3}{2})=0.58$.

From amplified regions, the distribution of $L_{\text {p}}^{\text {UMI}}$ can be decomposed as follows:

1 $$\begin{aligned} L_{\text {p}}^{\text {UMI}} = \log _2\left( c \times \frac{3}{2} + (1-c) \times \frac{2}{2}\right)\iff & {} 2^{L_{\text {p}}^{\text {UMI}}} = \frac{1}{2}c + 1\nonumber \\\iff & {} c = 2\left( 2^{L_{\text {p}}^{\text {UMI}}} - 1\right) \end{aligned}$$

The mean value of the distribution of $G_{\text {n}}^{\text {UMI}}$ is used in order to complete this Eq. (1) to estimate $c$.

The same decomposition is carried out considering the loss of a copy:

2 $$\begin{aligned} \begin{aligned} L_{\text {p}}^{\text {UMI}} = \log _2\left( c \times \frac{1}{2} + (1-c) \times \frac{2}{2}\right) \iff c = -2\left( 2^{L_{\text {p}}^{\text {UMI}}} - 1\right) \end{aligned} \end{aligned}$$

The mean value of the distribution of $D_{\text {n}}^{\text {UMI}}$ is used in order to complete this Eq. (2) to estimate $c$.

The algorithm output by default the mean value of this two independant estimates of $c$

## Results

### Comparison between read-depth and UMI-depth signals

**Fig. 3:**
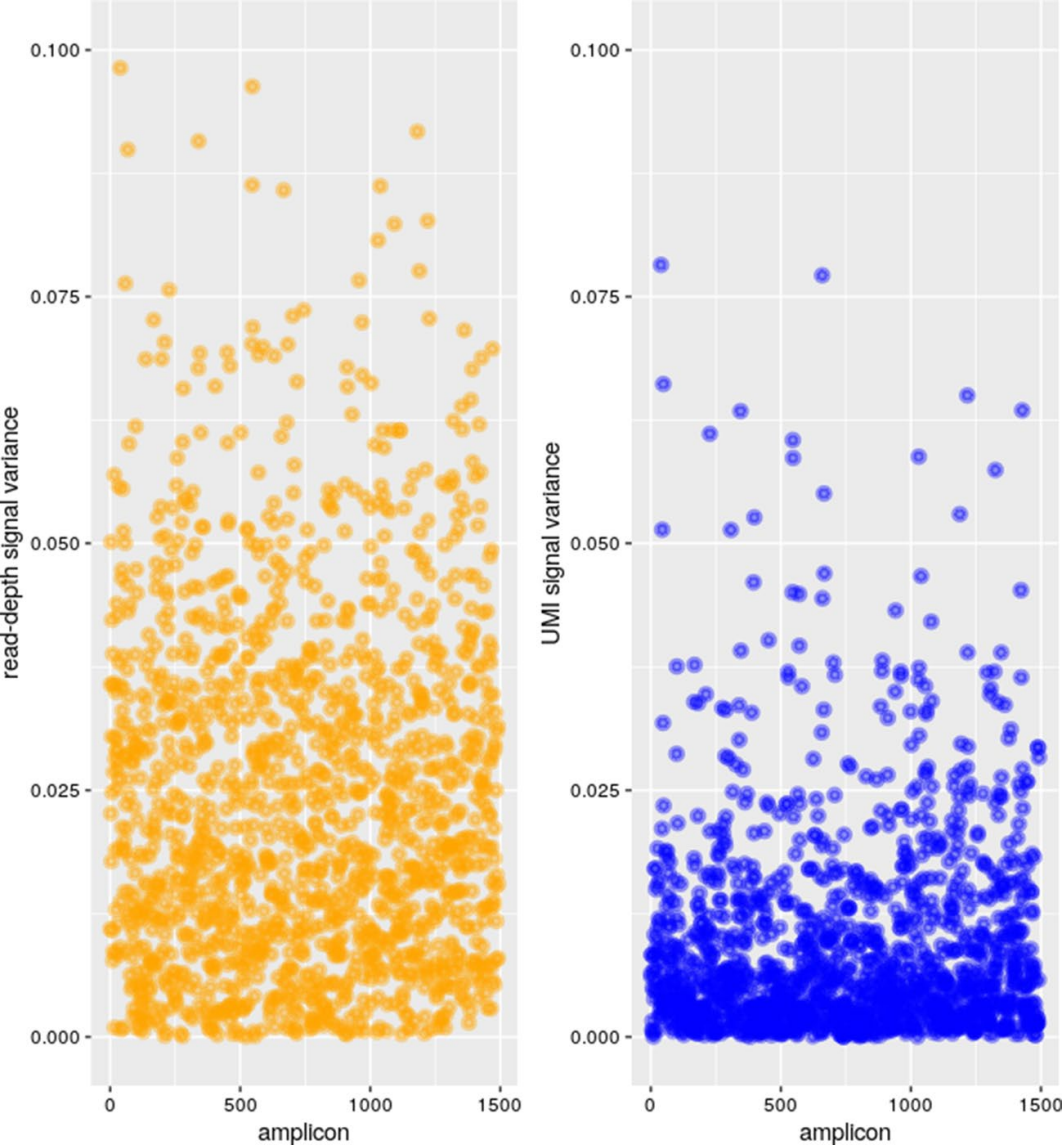
Variance of measurements within reference samples. The scatter plots represent the normalized read-depth counts on the left and the normalized UMI-counts on the right within control samples. Each targeted region is represented by a point

To allow the comparison between read-depth and UMI-depth signals, we extracted respective counts from our reference samples for each targeted region. Theses counts were normalized respectively by the mean read-depth/the mean UMI-depth to make samples comparable. Measured variances were significantly lower when taking into account the UMI-depth and not the read-depth (*p* value < 2.2e–16), as shown in Fig. 3.

### Construction of Pan-Lymphoma baseline

From mCNA quality control step, one control sample (CTL-22081) was excluded during pseudo-reference computation because of too high RMSD. The distribution of normalized UMI counts for this sample was clearly distinct from others as shown in the Additional file 1: Fig. S2. mCNA also detected 31 targeted regions not passing RMSD filters which were excluded. List of outliers and their characteristics are provided in Additional file 1: Table S2.

**Fig. 4:**
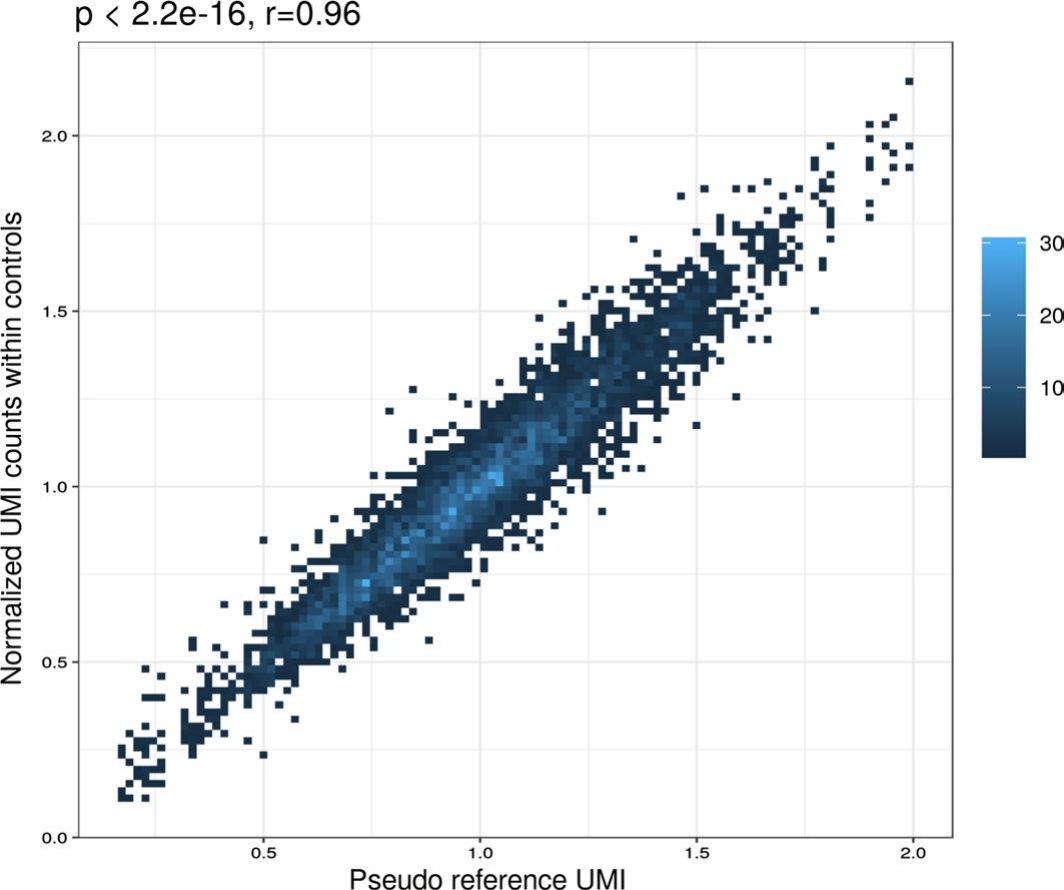
Correlation between normalized UMI count matrices and matched pseudo-reference vector. The scatter plot represents the normalized counts $C_{\text {p}}^{\text {UMI}}/U$ of $M^{\text {UMI}}$ matrices within control samples and the associated pseudo-reference vector $R^{\text {UMI}}$. Each targeted region is represented by a point. A Pearson correlation test is computed and correlation coefficient is displayed at the top of the figure

To validate our approach, we determined the correlation between normalized UMI count matrices of control samples and the computed pseudo-reference vector for each targeted region (Fig. 4). Signals were significantly and strongly correlated (r>0.96, *p* < 2.2e–16), which means that the computed pseudo-reference perfectly reflects the controls.

### Example of mCNA profile

**Fig. 5:**
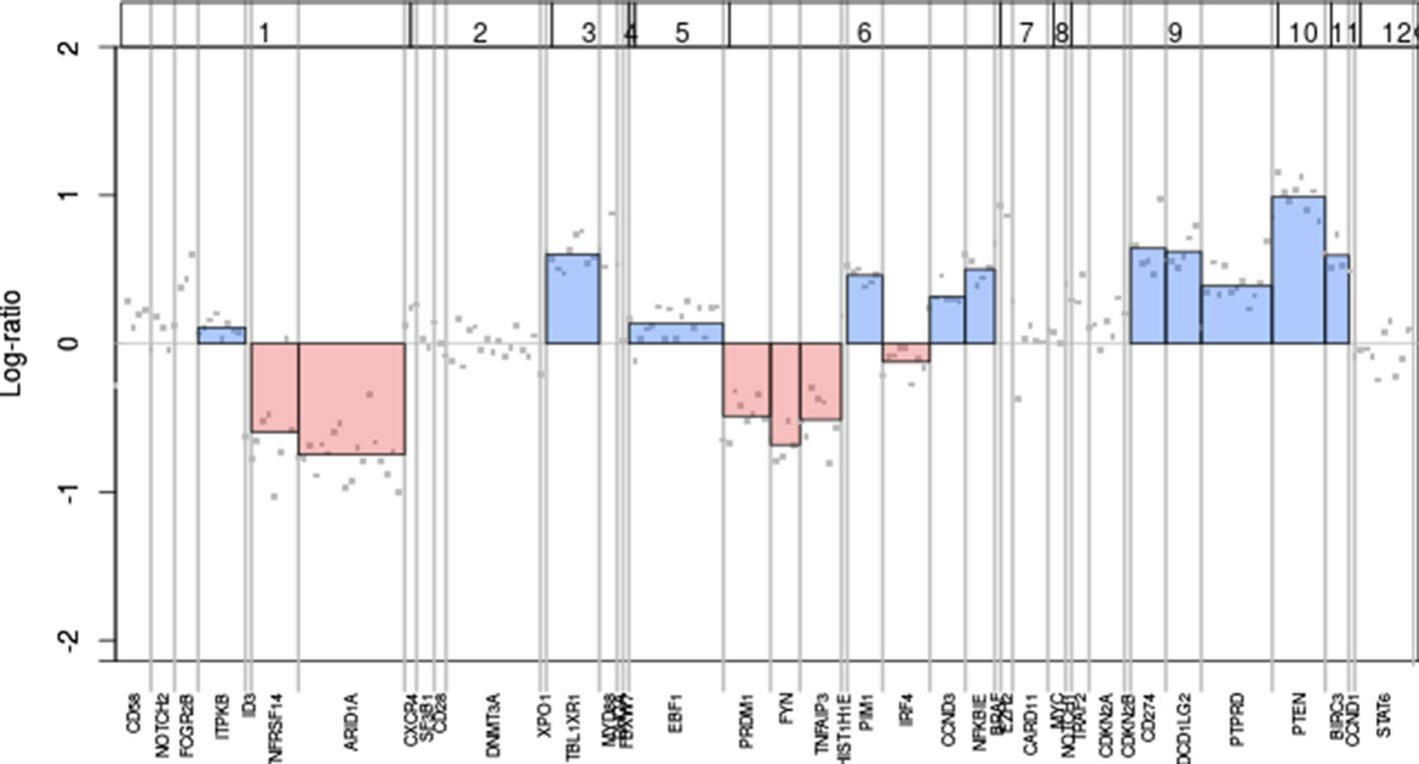
Example of mCNA profile. The top of the panel represents the chromosome location. Each vertical line delimits a gene. The log-ratio plot gives estimated log-ratios for the sample and the results of CBS segmentation. Blue/red segments represent respectively significant gain/lost segments. Gene names are displayed at the bottom of the graph

For each tested sample, mCNA generates a csv file summarizing by segment the measured data and the significance of the tests. A graph is also provided representing the log-ratios by region, the segmented signal and the results of the test. An example of profile is shown in Fig. 5.

### Comparison between mCNA and CGH data

**Fig. 6:**
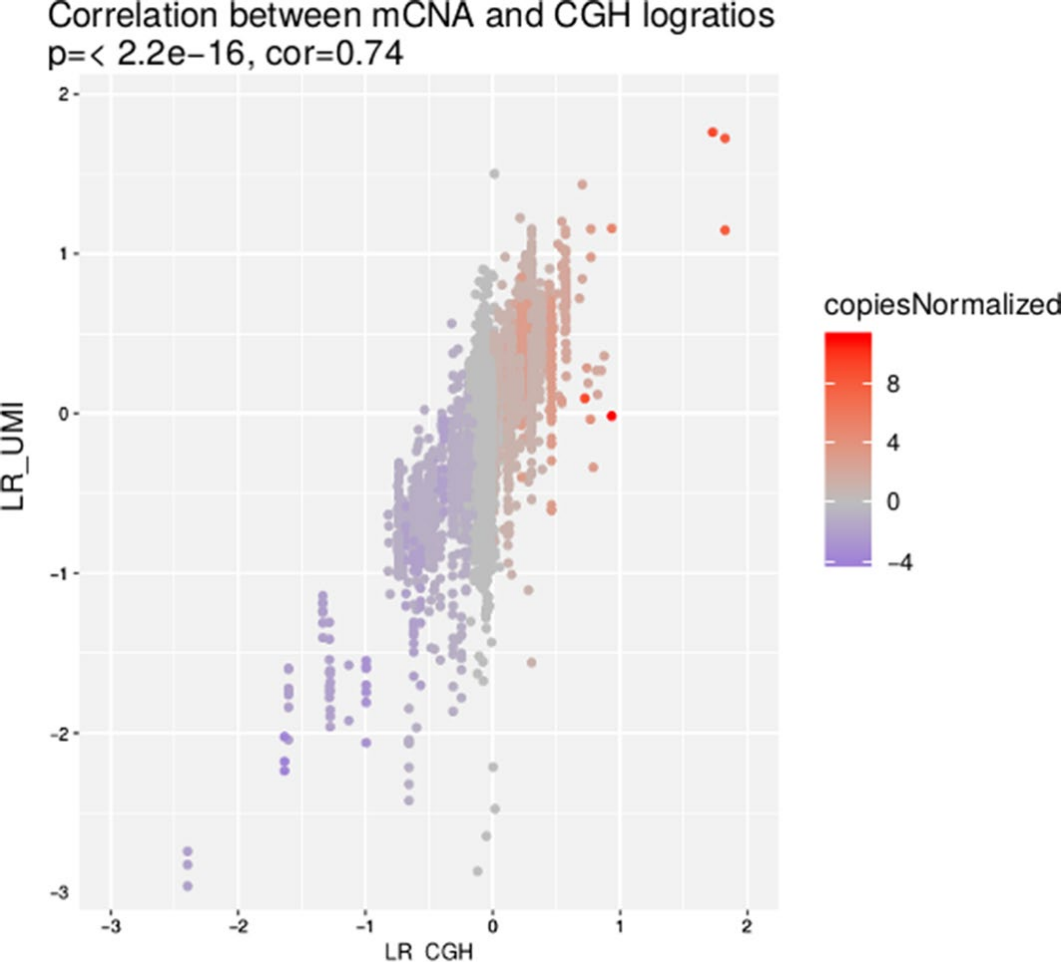
Correlation between mCNA and CGH log-ratios. Each point of the plot corresponds to a region of the Pan-Lymphoma Panel annotated by CGH results and estimated by mCNA. Colors show the number of gain or loss estimated by CGH data regarding the region. Pearson correlation coefficient is displayed at the top of the plot

In order to validate mCNA approach, we first compared CGH and NGS data (Fig. 6). We estimated log-ratios for each targeted region of the Pan-lymphoma panel using mCNA approach and then those estimated from CGH.

We observe a strong correlation between log-ratios of both technologies (r = 0.74). The majority of discrepancies are visible for $L_{\text {CGH}} = 0$ which may show a lack of sensitivity of CGH due to a lack of probe coverage.

To further our comparison, we extracted all predicted mCNA segments of our 22 tumor samples. These segments were then annotated with CGH results. 114/120 (95%) mCNA segments were predicted deleted by CGH and 175/221 (79%) were predicted as gain. 723/978 were predicted normal by mCNA and confirmed in CGH (74%), leading to an overall agreement between the two datasets of 83%.

### Robustness and sensitivity limit

To estimate theoretical sensitivity limit of mCNA approach, we first edited $M^{\text {UMI}}$ matrix of UMI count of one control sample (16,464) to introduce amplification of *XPO1*, gain of *IRF4*, heterozygous deletion of *CDKN2A* and homozygous deletion of *CDKN2B*. We applied an in silico dilution of these abnormal segments at 100%, 50%, 20%, 10% and 5% of tumor cells and applied mCNA to determine whether or not segments were significantly found after signal centering, segmentation and statistical test application. Results were summarized in Additional file 1: Fig. S4 and Additional file 1: Table S3. We found a strong correlation between expected and computed log-ratios (r = 0.99, *p* = 7.19e8-27) after signal centering and segmentation. mCNA was able to detect all in silico abnormal segments for tumor cell percentage between 10% and 100%. At 5%, only segments involving gain or loss of more than one copy were significantly found.

To confirm in silico results, REC-1 cell line was sequenced on two different runs to estimate the robustness of $L_{\text {p}}^{\text {UMI}}$ measurement using the Pan-Lymphoma panel. We found a strong correlation between the two replicates (r = 0.93, $p<0.001$) even if the sequencing depths were not the same (1851X/2217X). Details are provided in Additional file 1: Fig. S3.

30/31 segments were predicted as gains in both replicates (96.77%), 21/23 (91.30%) as normal and 17/18 (94.44%) as deleted, thus giving an average agreement of 94.17%. Discordant predictions result from segments having a low number of targeted regions and a small log-ratio variation.

**Fig. 7:**
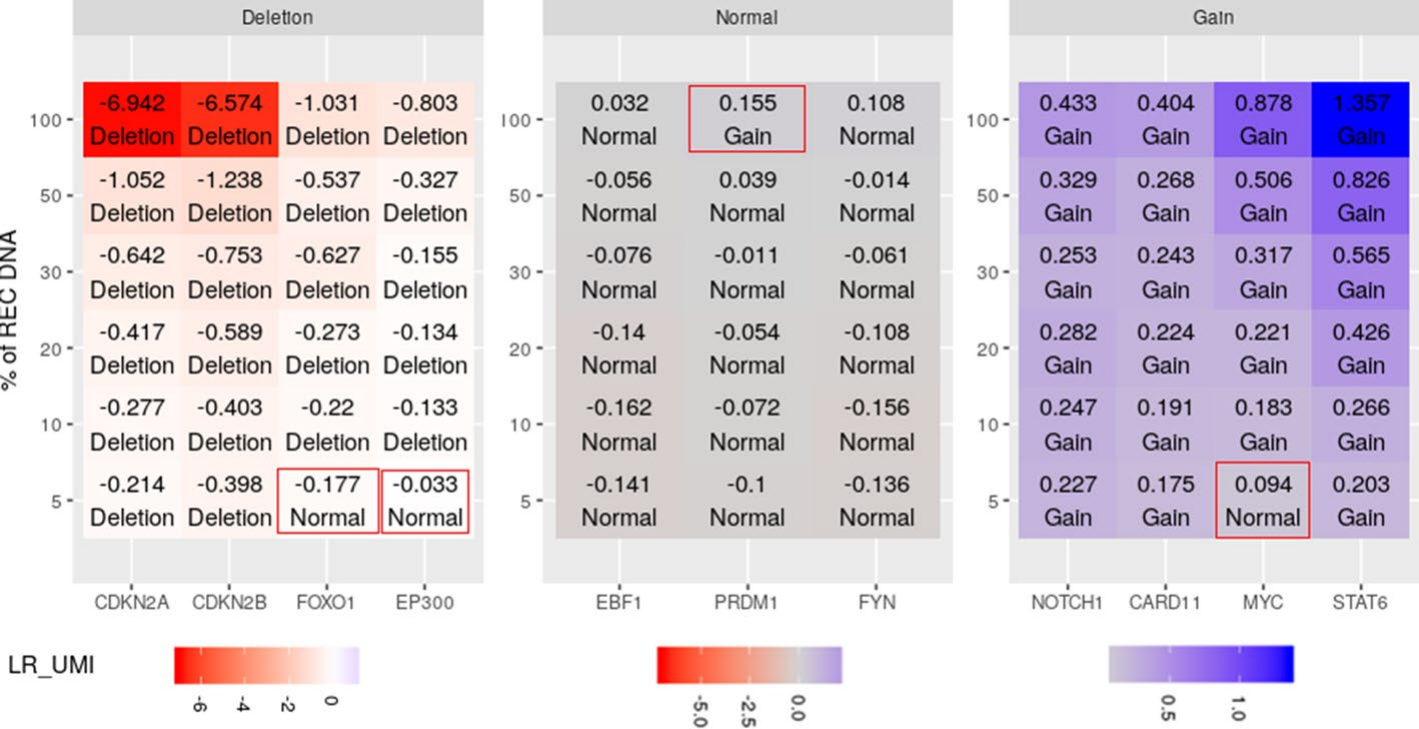
mCNA results of REC-1 cell line through dilutions. Each cell contains the log values obtained for the given gene and the final status of the segment given by mCNA

Finally, dilutions of REC-1 DNA were performed at 50%, 30%, 20%, 10% and 5%. REC-1 is a near-diploid cell line of male origin with a modal chromosome number of 45 and a polyploidy rate of 10%. Its karyotype is highly rearranged with approximately 5–6 derivative chromosomes in the karyology that have been described. Significant segments in this cell line were selected from the initial profile to evaluate the sensitivity of our approach through the different dilutions. Results seem consistent up to a threshold of 10% enrichment (Fig. 7). Above this threshold, the evaluation of tumor content seems consistent between expected and estimated percentage of tumor cells (r = 0.98) as shown in Additional file 1: Fig. S5.

### Comparison to read-depth algorithm

To assess mCNA’s analytical performance, we decide to compare our UMI-depth approach to the read-depth algorithm ONCOCNV [11] using REC-1 dataset. ONCOCNV was commonly used for the analysis of targeted sequencing panel of genes. It uses several normalization steps on read counts to erase library amplification biases such as library size, GC content of each region or amplicon length.

We hypothesized that the direct count of UMI could improve the limit sensitivity of ONCOCNV insofar as we have shown that the signal in UMI was less noisy than read counts. ONCOCNV results were generated for all REC-1 dilutions using the same control samples as those used to construct mCNA baseline.

**Fig. 8:**
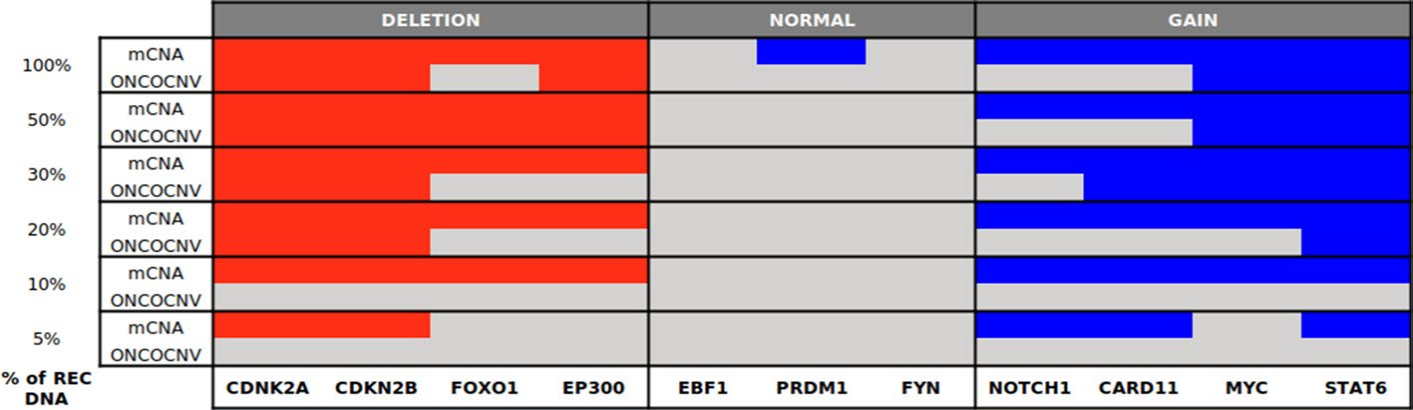
mCNA and ONCOCNV results of REC-1 cell line through dilutions. Each cell contains the calls obtained for the given gene

As expected, mCNA achieved much higher prediction accuracies than ONCOCNV as the percentage of tumor cells decreases (Fig. 8). Here, accuracy measures the proportion of genes with correctly annotated copy number status compared to the initial REC-1 profile: normal, gain or deletion. Considering the results of the algorithms from 100 to 10% of REC-1 DNA, the overall prediction accuracy fluctuated from 0.90 to 0.27 for ONCOCNV, while it was significantly higher for mCNA : 1.0 to 0.90. Interestingly, while mCNA results look consistent at 10%, ONCOCNV fails to detect heterozygous deletions of *FOXO1* and *EP300* at 30% of tumoral cell.

## Discussion

We proposed a new methodology to be used to detect copy number changes for targeted panels of genes using unique molecular identifiers. By changing the source of information from sequencing depth to UMI depth, mCNA provides a simple and robust methodology for the detection of CNV.

We demonstrated that using UMI-depth signal, and not read-depth signal, seems more robust in samples without abnormal copies. The algorithm uses a pool of reference samples to construct a pseudo-reference and includes a filtering step to automatically exclude samples and/or targeted regions with abnormal variance. We demonstrated that this in silico baseline profile reflects the reference samples and enables the estimation of CNV changes in unpaired tumor samples.

mCNA provides a strong estimation of log-ratios which correlates to our CGH dataset of 22 DLCBL samples. As we expected, the majority of discrepancies are visible for short breaks within genes probably due to a lack of probe coverage of Agilent SurePrint G3 4x180K microarrays. To avoid overestimation of breakpoints due to outlier values, mCNA provides a vector of weights to CBS segmentation function. We also recommend the use of at least 6 non-overlapping amplicons to properly estimate the state of a targeted region.

As we expected, we failed to detect CNA for samples that were highly contaminated by normal cells (less than 10% of tumor content). In this case, the noise in measurements is higher than the expected difference between measurements in the case of one CNV event. This observed threshold of 10% was confirmed by in silico simulation and also by sequential dilution of REC-1 cell line.

Our approach is designed to be used for targeted gene panels and thus doesn’t allow the combination of UMI-depth signal and B allele frequencies to improve the sensitivity of our CNV calling approach, as for analyses at the exome scale for example.

Another limitation of mCNA approach is the assumption that the majority of the signal corresponds to a diploid state. Polyploid profiles for example still remain challenging because the algorithm proceeds to center the signals. We recommend for panels targeting very frequently altered genes to deactivate this centering step.

Finally, mCNA gives the opportunity to obtain both the mutational and the copy number status at no additional cost. It helps in the interpretation of frequently altered genes, such as *TP53* for example, for which mutations are often associated with copy abnormalities.

## Conclusion

In this article, we present a new strategy to detect copy number changes for targeted panels of genes using UMI. mCNA is composed of four main steps: the construction of UMI count matrices, the use of reference samples to construct a pseudo-reference, the computation of log-ratios , the segmentation and finally the statistical inference of segmented breaks.

## Supplementary Information

**Additional file 1.** Supplementary Figures S1–S5, Supplementary Tables S1–S3.

## Data Availability

mCNA is available at https://gitlab.com/pierrejulien.viailly/mcna/ under MIT license. The datasets analysed during the current study are also available in mCNA data repository.

## Abbreviations

CNV: Copy number variation
UMI: Unique Molecular Identifiers
mCNA: Molecular Copy Number Alteration
LR: Log-ratio
NGS: Next Generation Sequencing
SNV: Single Nucleotide Variation
PCR: Polymerase chain reaction
RP: Read-pair
SR: Split-read
RD: Read-depth
RC: Read count
TSE: Targeted Sequencing Experiment
DLBCL: Diffuse Large B-Cell Lymphoma
PMBL: Primary Mediastinal B-cell Lymphoma
GSP: Gene Specific Primer
BED: Browser Extensible Data
BAM: Binary Alignment Map
RMSD: Root-Mean-Square Deviation
FDR: False Discovery Rate
