# Supplementary material for: Improving high-resolution copy number variation analysis from next generation sequencing using unique molecular identifiers
Source: BMC Bioinformatics. 2021 Mar 12;22:120. doi: 10.1186/s12859-021-04060-4 (PMC7971104; doi:10.1186/s12859-021-04060-4)
Supplement: Supplementary file 1 — Additional file 1. Supplementary Figures S1–S5, Supplementary Tables S1–S3. [file 12859_2021_4060_MOESM1_ESM.pdf]

SUPPLEMENTARY DATA

Supplementary Figures

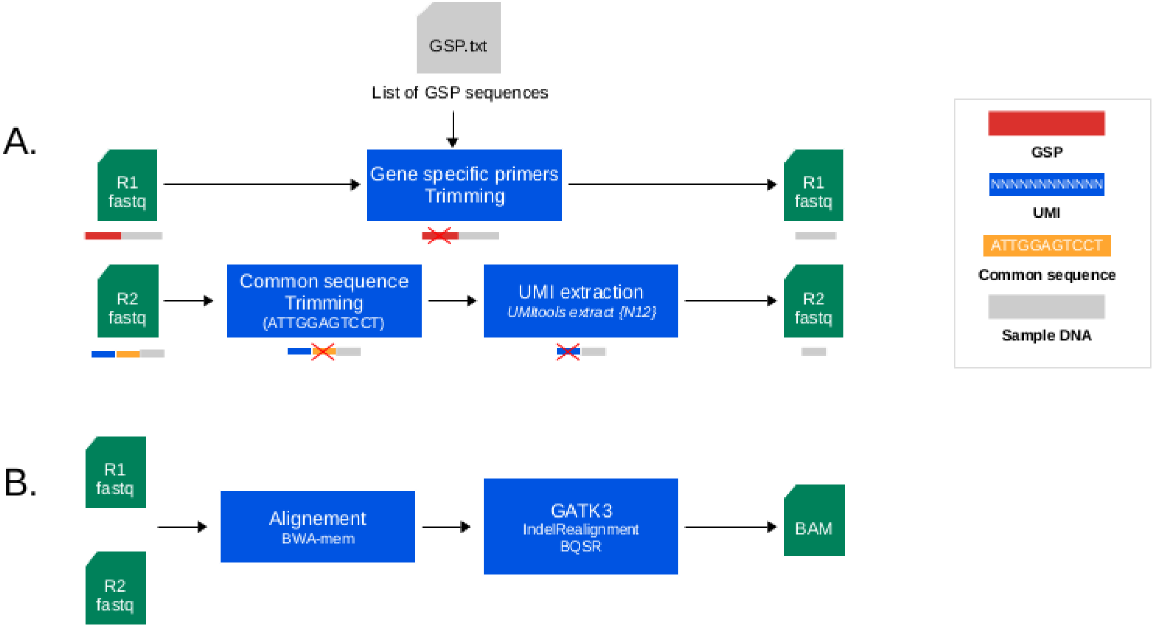

Figure S1 : Detailed bioinformatics processing of Qiaseq libraries. A. Fastq pre-processing. Gene Specific Primers (GSP) were eliminated from R1 fastq using the list of GSP sequences provided by the provider while creating the design. This step is performed by an in-house algorithm which compares the sequence start of each read and the list of GSP primers tolerating up to two sequencing errors. If no GSP primer was found, the read-pair is removed from the fastq. The common sequence (ATTGGAGTCCT) was removed from raw R2 reads directly by sequence comparison. UMI are then extracted from fastq sequences using UMI-tools and added at the end of each read name. **B. Alignment and GATK.** Reads were aligned against the reference genome (hg19) using BWA-mem and processed by GATK3 tools to create the Binary Alignment Map (BAM) file of each sample.

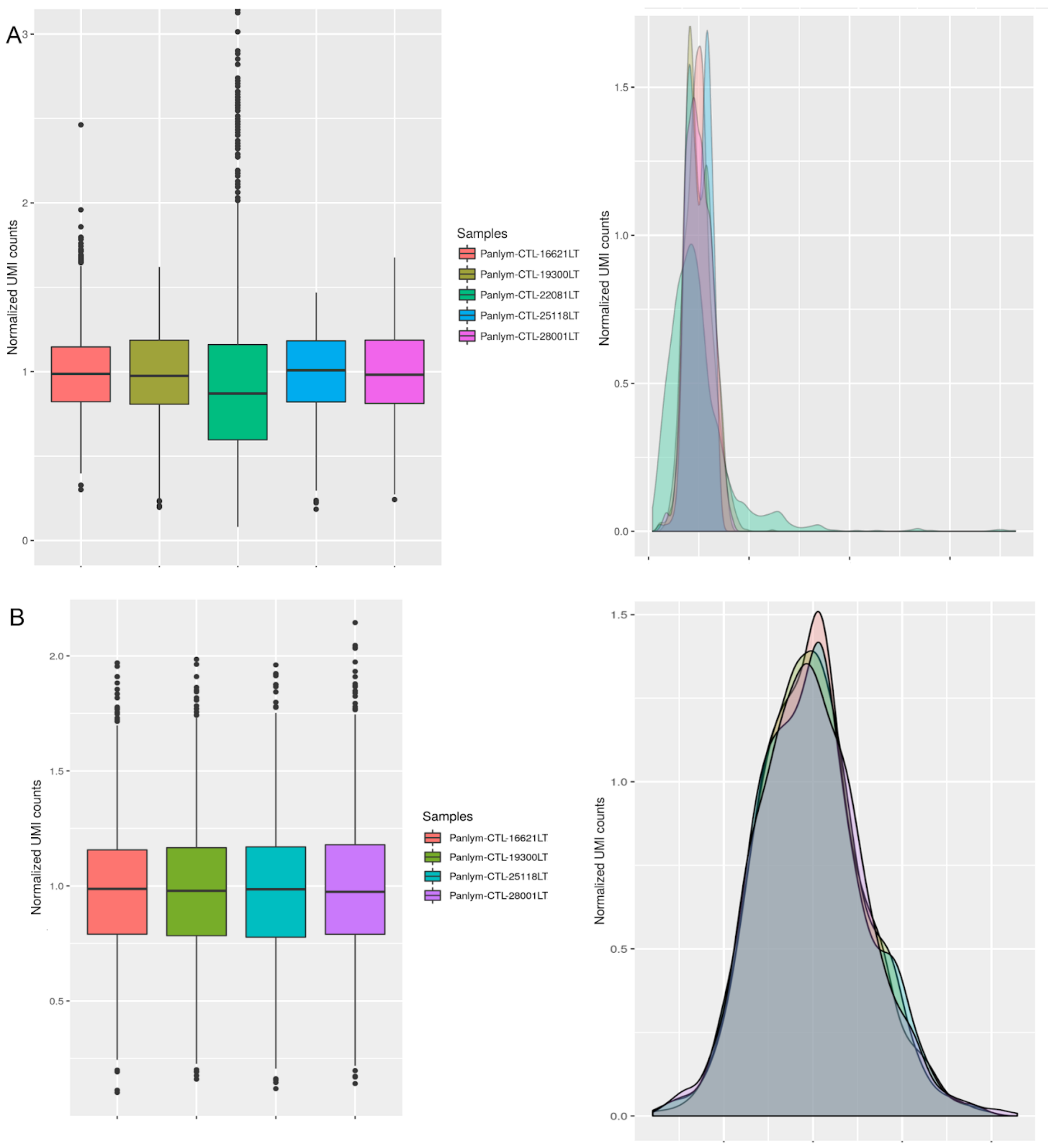

Figure S2 : PanLymphoma Panel : Distribution of Normalized UMI counts C^{UMI}/*U*. The left and right parts of the graph show respectively box plots and density distributions of C^{UMI}/*U* for 5 control samples. **A.** Distribution of C^{UMI}/*U* with outlier 22081 sample. **B.** Distribution of C^{UMI}/*U* within final list of samples used as reference.

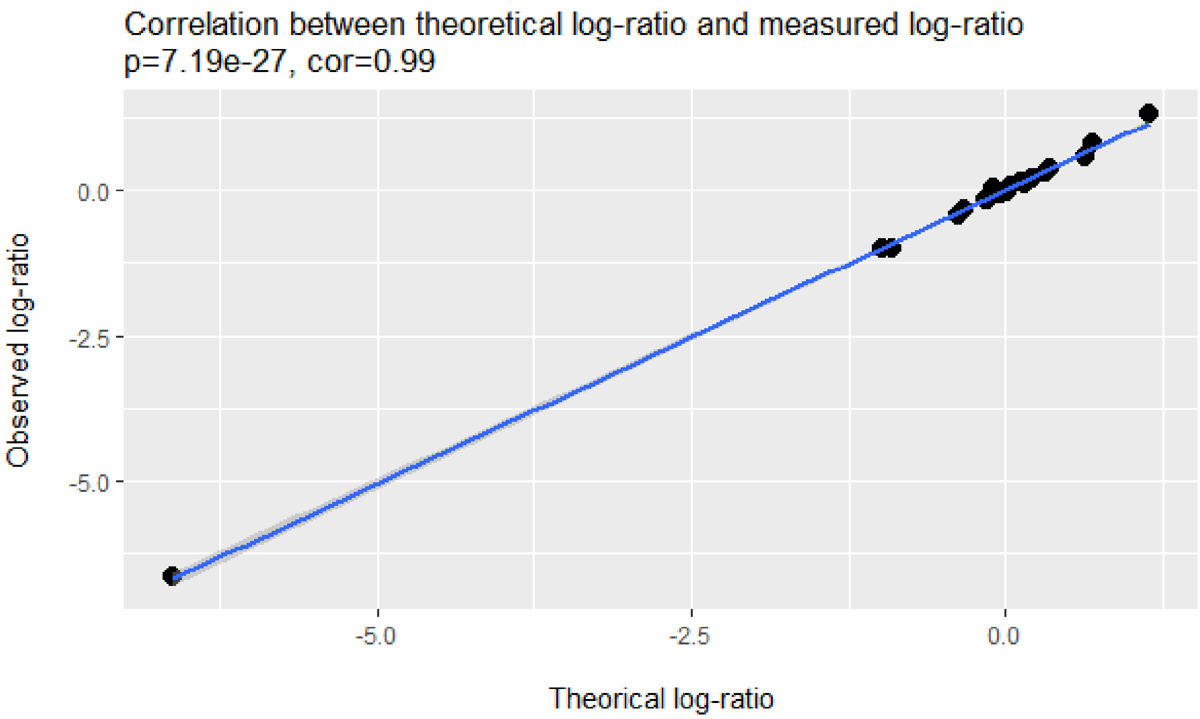

Figure S3 : Correlation between theoretical log-ratios and observed log-ratios in simulated dataset. Each point of the plot corresponds to L^{UMI} measurement for an abnormal segment. Amplification of *XPO1*, gain of *IRF4*, heterozygous deletion of *CDKN2A* and homozygous deletion of *CDKNBA* were introduced in 16464 control sample with a variable percentage of tumor cells (100%, 50%, 20%, 10% and 5%).

.

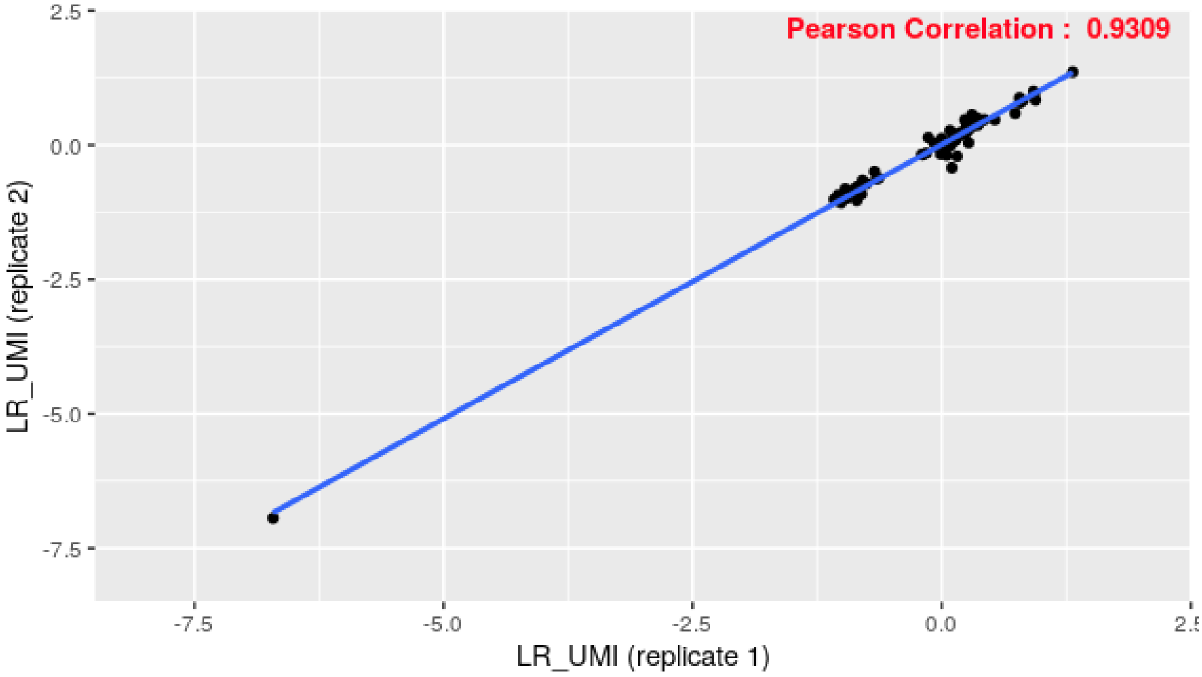

Figure S4 : Correlation between REC-1 replicates. Each point of the plot corresponds to L^{UMI} measurement for a segment between the two REC-1 replicates.

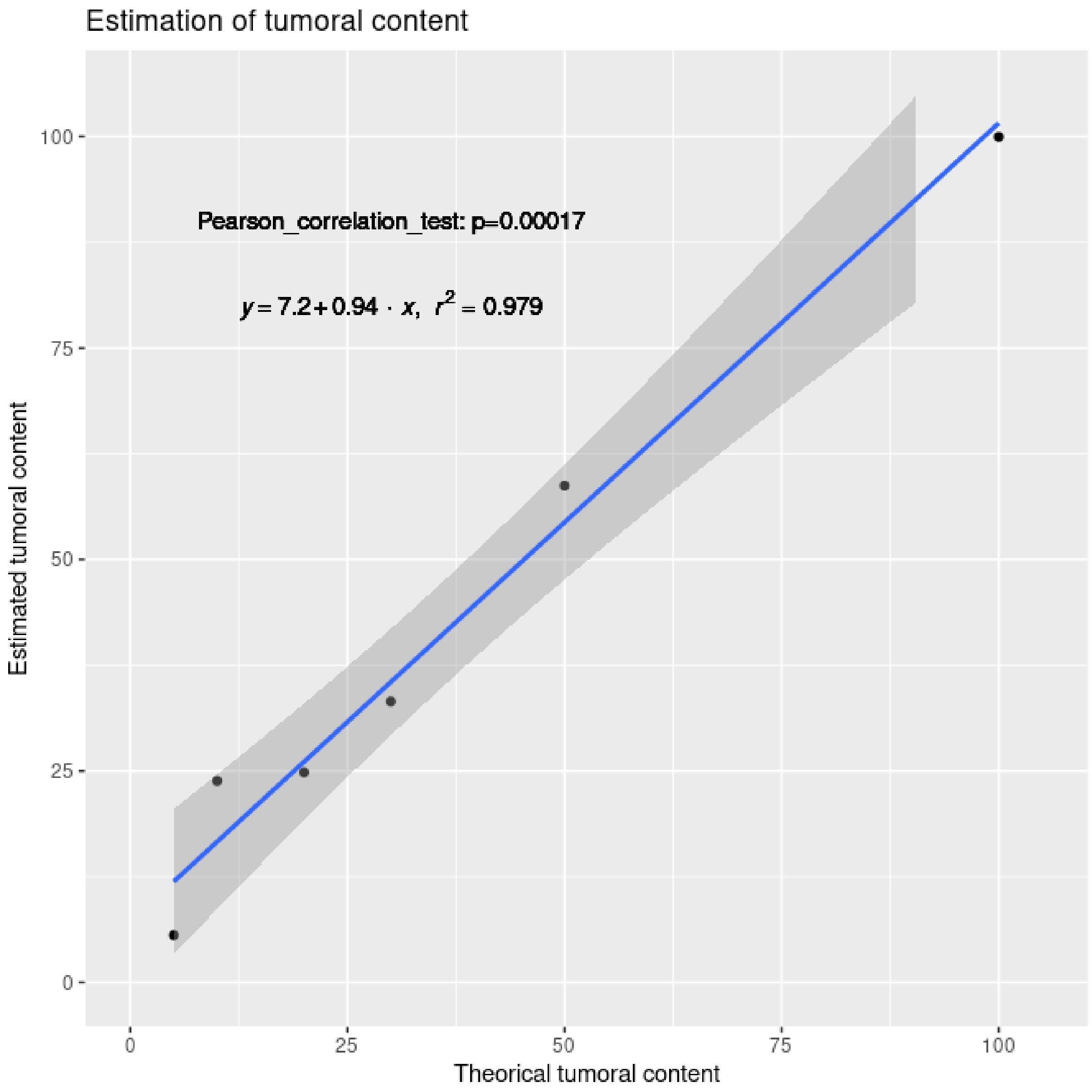

Figure S5 : Correlation between expected and estimated tumoral content in REC-1 dilutions.

Supplementary Tables

Table S1 : Pan-lymphoma Panel : Number of targeted regions per gene. Each one of these regions will allow the calculation of a log-ratio for the estimation of the number of copies for one gene.

Gene	Number of regions	Gene	Number of regions
ARID1A	84	IRF4	22
B2M	6	ITPKB	39

BCL2	8	JAK3	33
BCL6	25	KLF2	14
BIRC3	12	MEF2B	21
BRAF	2	MYC	16
BTG1	6	MYD88	8
BTK	3	NFKBIE	21
CARD11	16	NOTCH1	19
CCND1	4	NOTCH2	24
CCND3	15	PDCD1LG2	14
CD274	16	PIM1	14
CD28	6	PLCG1	62
CD58	17	PLCG2	19
CD70	10	PRDM1	32
CD79A	5	PTEN	22
CD79B	4	PTPN1	25
CDKN2A	28	PTPRD	26
CDKN2B	47	RHOA	2
chr14:106.3Mb	16	SF3B1	7
CIITA	65	SOCS1	8
CREBBP	102	SPEN	91
CXCR4	12	STAT3	5
DNMT3A	34	STAT5B	10
EBF1	39	STAT6	23
EP300	102	TBL1XR1	20
EZH2	5	TCF3	5
FBXW7	3	TET2	35
FCGR2B	14	TNFAIP3	31
FOXO1	22	TNFRSF14	16
FYN	11	TP53	24

GNA13	14	TRAF2	7
HIST1H1E	8	TRAF3	6
ID3	7	XPO1	2
IDH2	2		

Table S2 : Excluded regions : List of excluded regions and their characteristics.

interval	mean RMSE	%GC target	length target	strand primer	Gene Specific Primer sequence	%GC primer	len primer
chr1:226924916,226925042	0,29	76,38	127	0	GCCACTGCCGCTGCTACTATTCAG	58,33	24,00
chr1:27106519,27106644	0,41	55,56	126	1	AAACTGCCCAGTGTAGGAGTCCGTC	56,00	25,00
chr1:27106619,27106743	0,41	62,40	125	0	GGGCAGTTGGACCTATCTCCATACCC	57,69	26,00
chr1:27106723,27106850	0,40	54,69	128	1	CCGGGTTCTTTCGGTCACTGAGG	60,87	23,00
chr3:187462287,187462413	0,48	38,58	127	1	TGCCTTCGAGCCGAACCGAGATTT	54,17	24,00
chr3:187462403,187462520	0,51	58,47	118	0	CAAAAACAAAAACCCAAAGAGTTCGCTTGCATT	36,36	33,00
chr3:187463099,187463214	0,27	65,52	116	1	GGGTTCTTAGAAGTGGTGATGCAAGAAGTTTCTAG	42,86	35,00
chr3:187463199,187463324	0,34	48,41	126	0	GCGGCAGCAACAGCAATAATCACCT	52,00	25,00
chr3:187463299,187463421	0,33	52,03	123	1	TCCAAATCCGAGACGCTCTGCTTATGAG	50,00	28,00
chr3:187463375,187463501	0,35	59,06	127	1	GGAGCAGGCCATACCATCGTCTTG	58,33	24,00
chr4:106156848,106156967	0,25	47,50	120	0	CCAAGCGGAATCCCATCTAAAACGTAATGAG	45,16	31,00
chr6:138192353,138192467	0,59	42,61	115	1	TGTATCGGTGCATGGTTTTAAAATGATGAATGATCC	36,11	36,00
chr6:138192438,138192559	0,75	40,16	122	0	CGGAAAGCTGTGAAGATACGGGAGAGAAC	51,72	29,00

chr9:22004405,22004519	0,34	36,52	115	1	GGTGGGTAATTTGGTTTAAAAATACATGTTCATGGG	36,11	36,00
chr9:22004783,22004906	0,27	31,45	124	1	GCAAACTCAGTGCAAACGCCTAGATTG	48,15	27,00
chr9:22005544,22005660	0,35	55,56	117	1	TTCTGAGGGAAAGTGCATATGAAATCCTTGACTG	41,18	34,00
chr9:22005642,22005764	0,33	47,15	123	0	CTGGAGTGGGAGATTCATCCATCGGAAG	53,57	28,00
chr9:22005754,22005869	0,34	49,14	116	0	GAATGTCACACACTCCTAAATATCCCTGGAAATCC	42,86	35,00
chr9:22005965,22006080	0,35	73,28	116	0	GGTGGGAAATTGGGTAAGAAAATAAAGTCGTTGTG	40,00	35,00
chr9:22006067,22006196	0,38	73,08	130	0	AAGTCCACGGGCAGACGACCC	66,67	21,00
chr9:8341775,8341890	0,33	41,38	116	0	TGTCAGCTTCTGAATGTAGGCATACAAGTTTCTAG	40,00	35,00
chr11:102206801,102206913	0,42	34,51	113	0	CCTGGATAGTCTACTAACTGCCGGAATTATTAATGAAC	39,47	38,00
chr11:102206855,102206975	0,35	32,23	121	0	AGACACAGACGTCTTTACAAGCAAGAGAAC	43,33	30,00
chr16:3843381,3843499	0,49	52,94	119	1	CTGGTTCTACTGCTTCATGCTCATAAGTGTCA	43,75	32,00
chr16:3843497,3843621	0,61	47,20	125	0	AACCTCTCCGTTTGCTTGCTCTCGTC	53,85	26,00
chr16:3843539,3843651	0,53	38,94	113	1	TAATGAATATGACTTGGAACTCTGAGAGGTTAAAGTAT	31,58	38,00
chr16:3843591,3843708	0,51	30,51	118	1	CTCTCTGTGGTCGGTATTATCCATCAGCTTTTG	45,45	33,00
chr16:3843605,3843725	0,49	29,75	121	0	CTGTTGCAATTGCTTGTGTGGGTACAATTC	43,33	30,00
chr17:63010474,63010594	0,25	47,93	121	0	ATGTTCTCCGTGTTGATAGCAGTGGTGAAG	46,67	30,00
chr19:17945856,17945982	0,38	65,35	127	1	CATGGCAATGTCTCTGCCCGGAAG	58,33	24,00
chr19:17945908,17946029	0,38	65,57	122	0	CCAGGAACTTACTCTCCAGGCTTAACACA	48,28	29,00

Table S3 : Detailed results of *in silico* dilution. Each cell corresponds to L^{UMI} measurement for an abnormal segment. Amplification of *XPO1*, gain of *IRF4*, heterozygous deletion of *CDKN2A* and homozygous deletion of *CDKNBA* were introduced in 16464 control sample with a variable percentage of tumor cells (100%, 50%, 20%, 10% and 5%). The column "Significant segment" specifies whether mCNA was able to find the abnormal significant segment.

Gene	Type	percent of tumor cells	Theoretical log ratio	Observed log ratio	Significant segment
CDKN2A	-1	100	-1,00	-0,90	TRUE
CDKN2A	-1	50	-0,42	-0,38	TRUE
CDKN2A	-1	20	-0,15	-0,14	TRUE
CDKN2A	-1	10	-0,07	-0,07	TRUE
CDKN2A	-1	5	-0,04	0,01	FALSE
CDKN2B	-2	99	-6,64	-6,63	TRUE
CDKN2B	-2	50	-1,00	-0,98	TRUE
CDKN2B	-2	20	-0,32	-0,33	TRUE
CDKN2B	-2	10	-0,15	-0,17	TRUE
CDKN2B	-2	5	-0,07	-0,03	TRUE
IRF4	+1	100	0,58	0,63	TRUE
IRF4	+1	50	0,32	0,33	TRUE
IRF4	+1	20	0,14	0,12	TRUE
IRF4	+1	10	0,07	0,04	TRUE
IRF4	+1	5	0,04	-0,09	FALSE
XPO1	+4	100	1,31	1,15	TRUE
XPO1	+4	50	0,81	0,70	TRUE
XPO1	+4	20	0,38	0,35	TRUE

XPO1	+4	10	0,20	0,22	TRUE
XPO1	+4	5	0,10	0,15	TRUE
